# A Node-Expressed Transporter OsCCX2 Is Involved in Grain Cadmium Accumulation of Rice

**DOI:** 10.3389/fpls.2018.00476

**Published:** 2018-04-11

**Authors:** Xiaohua Hao, Meng Zeng, Jian Wang, Zhongwen Zeng, Jiali Dai, Zijing Xie, Yuanzhu Yang, Lianfu Tian, Liangbi Chen, Dongping Li

**Affiliations:** ^1^Hunan Province Key Laboratory of Crop Sterile Germplasm Resource Innovation and Application, College of Life Science, Hunan Normal University, Changsha, China; ^2^College of Life and Environmental Science, Hunan University of Arts and Science, Changde, China; ^3^Yuan Longping High-Tech Agriculture Co., Ltd., Changsha, China; ^4^Key Laboratory of Chemical Biology & Traditional Chinese Medicine Research, Ministry of Education of China, Hunan Normal University, Changsha, China

**Keywords:** *Oryza sativa*, cadmium accumulation, translocation, distribution, transporter

## Abstract

Excessive cadmium (Cd) accumulation in grains of rice (*Oryza sativa* L.) is a risk to food security. The transporters in the nodes of rice are involved in the distribution of mineral elements including toxic elements to different tissues such as grains. However, the mechanism of Cd accumulation in grains is largely unknown. Here, we report a node-expressed transporter gene, *OsCCX2*, a putative cation/calcium (Ca) exchanger, mediating Cd accumulation in the grains of rice. Knockout of *OsCCX2* caused a remarkable reduction of Cd content in the grains. Further study showed that disruption of this gene led to a reduced root-to-shoot translocation ratio of Cd. Moreover, Cd distribution was also disturbed in different levels of internode and leaf. OsCCX2 is localized to plasma membrane, and *OsCCX2* is mainly expressed in xylem region of vascular tissues at the nodes. OsCCX2 might function as an efflux transporter, responsible for Cd loading into xylem vessels. Therefore, our finding revealed a novel Cd transporter involved in grain Cd accumulation, possibly via a Ca transport pathway in the nodes of rice.

## Introduction

Cadmium (Cd) is a very toxic heavy metal element and is harmful to humans’ health, when accumulated in the body. Cd impacts DNA synthesis and cell proliferation (von Zglinicki et al., 1992; Misra et al., 2003). Cd exposure enhances oxidative stress, inhibits function of DNA repair systems, and finally leads to cellular DNA damage (Jin et al., 2003; Waisberg et al., 2003; Lutzen et al., 2004). Over-accumulation of Cd can cause serious health problems, such as itai-itai disease (Horiguchi et al., 1994; Takahashi et al., 2012a). Besides bone lesion, Cd also produces damage to lung, kidney, liver, or even testes (Matovic et al., 2011, 2015). Recent studies revealed association of Cd intoxication with the carcinogenesis and development of diverse cancers, including renal cancer (Il’yasova and Schwartz, 2005; Huff et al., 2007; Song et al., 2015), prostate cancer (Vinceti et al., 2007; Rapisarda et al., 2018), breast cancer (Van Maele-Fabry et al., 2016), testis cancer (Goyer et al., 2004), bladder cancer (Feki-Tounsi and Hamza-Chaffai, 2014), and pancreatic cancer (Buha et al., 2017). As rice is the staple food for most Asians, rice becomes the major dietary Cd source for Asians. Therefore, lowering grain Cd content to safety level is very pivotal for people living in Cd exposure areas to alleviate Cd damage (Watanabe et al., 2004; Moon et al., 2013). Recently, significant progress has been made in elucidating molecular mechanisms of Cd uptake, transport, and accumulation in rice (Clemens et al., 2013). Cd was shown transported by utilizing some essential element transporters, such as some Zn, Mn, and Fe transporters. For example, OsNramp5 is a root-expressed, plasma-membrane-localized Mn transporter, responsible for Cd uptake (Sasaki et al., 2012). The OsNramp5 knockout mutant, *osnramp5*, greatly decreased Cd uptake by the root from the environment, and also significantly reduced grain Cd accumulation (Ishimaru et al., 2012a; Sasaki et al., 2012; Takahashi et al., 2014; Yang et al., 2014). In addition to OsNramp5, another plasma-membrane-located OsNramp member, OsNramp1, functioning as an iron transporter, mediates Cd uptake in the root (Takahashi et al., 2011a). Two other iron transporters, OsIRT1 and OsIRT2, were reported to be involved in Cd uptake as well (Nakanishi et al., 2006; Clemens et al., 2013; Yang and Zhang, 2016; Yang et al., 2016). OsHMA3 is able to sequester Cd into vacuoles of root cells, thereby alleviating toxicity to the cells (Ueno et al., 2010; Miyadate et al., 2011; Wu et al., 2015). Mutation of some key sites of *OsHMA3* gene resulted in a higher Cd accumulation in shoot of rice including grains (Ueno et al., 2010, 2011; Miyadate et al., 2011; Yan et al., 2016; Uraguchi et al., 2017), while over-expressing of *OsHMA3* caused decreased Cd accumulation in the grains (Ueno et al., 2010).

The nodes of graminaceous plants including rice contain complex, well-organized vascular systems and play important roles in the distribution of multiple mineral elements. Some node-expressed transporters are involved in ion distribution by mediating intervascular transfers in the nodes (Yamaji and Ma, 2014, 2017; Xu et al., 2017). The node-expressed *OsFRDL1* functions as a citrate efflux transporter, involved in the distribution of Fe via solubilizing Fe deposited in the apoplastic part of the upper nodes of rice (Kobayashi et al., 2014; Yokosho et al., 2016). *SPDT* is also a node-expressed plasma-membrane-located phosphorus (Pi) transporter, mediating the distribution of Pi to the rice grains, where knockout of *SPDT* led to reduced Pi accumulation in the grains but with increased Pi levels in the leaves of *spd* mutant (Yamaji et al., 2017). OsZIP3 functions as a Zn transporter in the node, responsible for controlling the allocation of Zn to the developing tissues in rice (Sasaki et al., 2015). Suppressed expression of this gene resulted in decreased Zn levels in shoot meristem and elongation zone, but elevated Zn accumulation in mature leaves in the RNAi plant. However, the root-to-shoot translocation did not affect (Sasaki et al., 2015). Several node-expressed transporters have been identified to transport Cd. Among them, OsHMA2 is expressed at the phloem of the nodes, responsible for the Zn and Cd delivery to developing tissues of rice. OsHMA2 is also located in root pericycle cells, mediating root-to-shoot translocation of Cd. Knockout of *OsHMA2* led to remarkably lowered Cd accumulation in the shoots and grains of the mutant compared to the wild type. Interestingly, both *OsHMA2* mutant and overexpression plants result in reduction of Cd in the leaves (Satoh-Nagasawa et al., 2012; Takahashi et al., 2012a; Yamaji et al., 2013). Another transporter, OsLCT1, has been shown to mediate phloem Cd transport in nodes and leaf blades of rice. Knockdown of this gene resulted in decreased Cd in phloem sap and in the grain (Uraguchi et al., 2011). However, the underlying mechanism for Cd accumulation in grains of rice as a whole remains largely unknown.

Cation/Ca exchangers (CCXs) belong to the cation/calcium (CaCA) superfamily, which is widely spread from bacteria to higher animals and plants. The CaCA transporters are able to exchange calcium (Ca) with other cation species such as H^+^, K^+^, or Na, where Ca transport is against membrane electrochemical gradient (Cai and Lytton, 2004; Emery et al., 2012). In *Arabidopsis, AtCCX1* is highly induced during leaf senescence. Knockout of *AtCCX1* and *AtCCX4* produced stay-green leaf, whereas overexpression of *AtCCX1* accelerated leaf senescence. Under Ca deficiency, both *ccx1* and *ccx1ccx4* seedlings displayed obvious growth impairment, implying that AtCCX1 may regulate leaf senescence via Ca signaling (Li et al., 2016). AtCCX3 and AtCCX4, localized to tonoplasts, showed capability in regulation of H^+^-dependent K^+^ as well as Na and Mn transportation (Morris et al., 2008). Transgenic tobacco (*Nicotiana tabacum*) overexpressing *AtCCX3* showed lesions in leaves and accumulated high levels of cations (Morris et al., 2008). AtCCX5 has been shown to act as a K transporter in the yeast; however, its biological function in planta remains unknown (Zhang et al., 2011). In rice, the genome encodes four putative CCX members, designated as *OsCCX1* to *OsCCX4* (Emery et al., 2012; Garg et al., 2012; Singh et al., 2014). All four members share highly conserved motifs GNG(A/S) PD and (G/S)(N/D) SxGD, originally detected in NCKX6 (Cai and Lytton, 2004). OsCCX2 was first classified as a cysteine-rich peptide family and designated as OsCDT1, which was shown to enhance Cd tolerance in yeast and *Arabidopsis* (Kuramata et al., 2009; Matsuda et al., 2009). Recently, *OsCCX2* was heterologously identified to be a K^+^-independent Ca^2+^/cation transporter in yeast (Yadav et al., 2015). However, no *OsCCX* has been functionally characterized in rice.

To understand the mechanism of the node function in mediating cation translocation and distribution, the node-expressed transporter genes were screened by transcriptome approach, and nine candidate transporter genes were selected for further study including two Cd characterized gene *OsHMA2* and *OsLCT1* and seven function-unknown genes such as *OsCCX2*, ABC transporter Os04g0459000, etc. Subsequently, the knockout mutants of these genes were generated by CRISPR/cas9 editing method. The grain Cd content of the mutants was thereafter detected after harvesting from Cd-contaminated paddy field. Here, we report a node-expressed transporter gene, *OsCCX2* (Os03g0656500), involved in Cd accumulation in the grains of rice. Knockout of *OsCCX2* resulted in a significant Cd reduction in the grains. OsCCX2 is localized in plasma membrane and plays a role at the nodes in mediating Cd translocation and distribution.

## Materials and Methods

### Plant Materials and Growth Conditions

Rice seeds were rinsed in distilled water at 30°C for 2 days in an incubator. The germinating seeds were then transferred to grow in a hydroponic container. The hydroponic solution (pH 5.7), suggested by Tezuka et al. (2010), contains 0.18 mM CaCl_2_, 0.23 mM MgSO_4_, 0.09 mM Na_2_HPO_4_, 0.18 mM NH_4_NO_3_, 0.14 mM K_2_SO_4_, 0.09 mM SiO_2_, 22.5 μM Fe(III)-EDTA, 9.2 μM H_3_BO_3_, 2.3 μM MnSO_4_, 0.78 μM CuSO_4_, 0.77 μM ZnSO_4_, and 0.5 μM (NH_4_)_6_Mo_7_O_24_. The seedlings in the hydroponic container were cultured for 10 days in chamber (14 h light at 28°C, 10 h dark at 25°C) before being used for different treatments. Then, the seedlings were transplanted to other hydroponic containers with nutrient solution containing different (0, 0.1, and 5 μM) CdCl_2_ and continue to be cultured for another 7 days. The nutrient solution with or without CdCl_2_ was renewed every 7 days. Each experiment was replicated in three times.

Fifteen-day-old seedlings of the wild type, ccx2-1 and ccx2-2 plants were transplanted according to the randomized complete block design method in paddy soil (Cd, 3.9 or 1.2 mg/kg) at the middle of June of three consecutive years from 2015. The plants were harvested and used for Cd determination.

### Semi-quantitative RT-PCR and q-RT-PCR

Total RNA samples from different parts were prepared by using the Trizol reagent (Invitrogen), and the genomic DNAs in the samples were eliminated with DNase (Invitrogen). The cDNA samples were made by the Reverse Transcriptase kit (Invitrogen) with anchored oligo (dT18). The OsCCX2 was amplified using primers in Supplementary Table S1 (OsCCX2-qPCR). For semi-quantitative PCR, 0.5 μl cDNA template was used with a total reaction volume of 20 μl PCR reaction system (Takara), and followed by different reaction cycles (Li et al., 2015). For q-RT-PCR, 1.5 μl cDNA template (the template was diluted 10 times compared with semi-quantitative PCR) was used with a total reaction volume of 30 μl reaction system (Promega, SYBR Green I), and then the reaction was completed in Quantstudio 5 (Thermo Fisher Scientific). The experiments were repeated three times independently.

### Genome Editing

We used the RNA guided genome editing technology (CRISPR-Cas9) to target the *OsCCX2* gene in *Nipponbare*. Target sites of 20 nucleotides were selected following the criteria described by Jin (Miao et al., 2013). The trinucleotide NGG (PAM site) at the 3′ end of the spacer was an essential criterion in target site selection. The two sgRNAs were designed to target two sites, respectively, in *OsCCX2* genes. The two targeted site spacers are: GTTTTATGGCGCTCCTGCGCAGG and: CCCTCGCCGCCTGACAATCCCGG. (The PAM sites were underlined.)

### Plant Transformation

The genetic transformation of *Nipponbare* cells was performed according to the procedures suggested by Toki et al. (2006) with some modifications. Briefly, the dehulled seeds were sterilized with 70% ethanol and 2.5% sodium hypochlorite for 1 and 15 min, respectively, and washed five times with sterile water. The sterilized seeds were then cultured on N_6_D medium under continuous light at 30°C for 5–7 days to induce calli. At the same time, *Agrobacterium* cells EHA105 harboring CRISPR-Cas9-CCX2 were grown on AB medium plates 50 mg/L spectinomycin at 30°C for 3 days in the dark. Then the *Agrobacterium* cells were collected and diluted in AAM liquid medium to yield a suspension of approximately 0.1 (OD_600_). Subsequently, the calli were immersed in this suspension and shaken gently for 90 s. These calli were dried on sterilized filter papers in a laminar flow bench, and were transferred onto 2N_6_-AS solid medium plates at 25°C for 3 days in the dark. Then the calli were washed six times to remove *Agrobacterium* cells, dried, and transferred to N_6_D solid medium (50 mg/L hygromycin and 400 mg/L carbenicillin). The plates were placed at 30°C with continuous light until the fresh calli occurred within 2–4 weeks. Then the fresh calli were transferred to RE-III medium to generate plantlets, which were further transferred to HF medium to induce root formation.

### Analysis of Metal Concentration in Different Organs

The hydroponic seedlings were washed three times with deionized water and divided into shoot and root tissues. The plants grown in paddy soil were separated into different organs including brown rice, husk, rachis, each leaf, each node, and root. These materials were dried at 105°C for 12 h and 70°C for 3 days in an oven, and then each sample was weighed and digested with HNO_3_–HClO_4_ (4:1 v/v) mix solution at 160–180°C. Graphite furnace atomic absorption spectrometry (GFAAS) or inductively coupled plasma mass spectrometry (ICP-MS) was used to determine the metal concentrations of each samples (Su et al., 2014).

### Cd Tolerance Analysis in Yeast Cells

The *Saccharomyces cerevisiae* strain *ycf1* (*MAT alpha, Δtrp1, Δhis3, Δleu2, Δura3*) is hypersensitive to CdCl_2_ due to its *ycf1* deletion (Li et al., 1996; Kuramata et al., 2009), and the wild type strain BY4741 (genotypic markers: *MATa his2Δ0met15Δ0 ura3Δ0*) was provided by Dr. Zhang (Yuan et al., 2012). The vector pYES2 or the pYES2-*OsCCX2* recombinant vector was transformed into them. The *ycf1* cells expressing pYES2-*OsCCX2* or empty vector pYES2 were spread onto synthetic Cd-containing drop-out (SG) medium (0, 10, and 20 μM CdCl_2_) lacking uracil plates (SG-Ura) and grown at 30°C for 3 days (Guthrie and Fink, 2002).

For Cd content analysis, the BY4741 transformants expressing pYES2-*OsCCX2* or empty vector pYES2 were grown in SG-Ura liquid medium containing 1, 2, 5, and 10 μM CdCl_2_ to the log phase, then the cells were collected by centrifugation, and then washed three times with deionized water. After drying at 55°C, the cellular pellets were digested with HNO_3_–HClO_4_ (4:1 v/v) mix solution at 120°C, and then the Cd content was analyzed by GFAAS.

### Separation of Xylem Sap and Cell Sap

To collect xylem sap, the mutant and wild type rice were grown in paddy soil (1.2 mg/kg Cd) till the grain-filling initiation stage. First, a cut was made at 2 cm above the first node with a razor blade and the cut surface was masked by an eppendorf tube (1.5 ml) filled with a small piece of absorbent cotton for 12 h. Then the sap was collected by centrifugation, and the volume of the collected sap was quantified with the Cd content determined subsequently (Lancilli et al., 2014).

The cell sap separated as suggested by Weigel and Jager (1980) and (Wang et al., 2008) with minor modifications. The shoot and root tissues were collected at 1, 2, 3, 4, 5, 6, and 7 days after Cd exposure started. Shoot and root were homogenized in a solution containing 0.25 M sucrose, 50 mM Tris-HCl (pH 7.5), and 1 mM dithiothreitol. The resulting brei was centrifuged at 6000 rpm for 10 min and the supernatant solutions were collected for further analysis. All steps were performed at 4°C.

### Histochemical Analysis of *OsCCX2* Expression

To investigate the tissue specificity of *OsCCX2* expression, the *OsCCX2* 2300bp promoter sequence (-2300 to -1 bp upstream from the initiation codon ATG) was subcloned into the binary vector pCambia 1300 (containing β-glucuronidase gene), and the recombinant plasmid was transformed into rice (*Nipponbare*) using *Agrobacterium*-mediated transformation method system (Toki et al., 2006). The transgenic plants containing the *Pro_CCX2_*:GUS element were stained for GUS signal detection according to the procedures (Jefferson et al., 1987). The plants were incubated overnight at 37°C in the staining solution containing 0.5 mM X-Gluc. After staining, plants were destained in 75% ethanol for 12 h, and renewed with 75% ethanol every 2 h. Then the plant mounted for photography or paraffin section.

### Paraffin Sections of Node

After the GUS transgenic rice were destained, the node samples were washed with 70% alcohol for three times (2 h for each time), and then the nodes were gradually dehydrated in increasing alcohol concentration gradient till 100% (2 h for each alcohol concentration). Node samples were immersed in 50% xylene and 50% ethanol overnight, and continued to increase xylene proportion till to 100% xylene. Next, the wax immersion of samples was performed in 50% xylene and 50% paraffin at 42°C overnight and continued at 60°C for 4 h in incubator. Subsequently, samples were transferred into 100% paraffin at 60°C for 8 h and the paraffin was renewed every 2 h. Finally, the samples were embedded in paraffin, and sliced with in a Leica RM2015 rotary microtome (Li et al., 2015).

### Subcellular Localization of *OsCCX2*

The *OsCCX2* cDNA fragment was amplified by RT-PCR from *Nipponbare* cDNA, and cloned into pCambia1302 in frame to generate the C-terminal GFP-tagged fusion protein, and then the recombinant vector was transformed into rice. The root of the transgenic plant expressing the *Pro*_35S_:OsCCX2-GFP fusion protein was visualized under confocal microscope (LSM, 710 META, Zeiss). The dye FM4-64 (a plasma membrane fluorescent marker) was used to stain plasma membrane of the root cells for co-localization. The pCambia2300 vector was used to express transiently OsCCX2-GFP fusion protein in *Arabidopsis* mesophyll protoplasts for localization. GFP excitation wavelength was 488 nm, and emission wavelength for detection was between 500 and 575 nm.

## Results

### *OsCCX2* Is Preferentially Expressed at the Nodes of Rice

**FIGURE 1:**
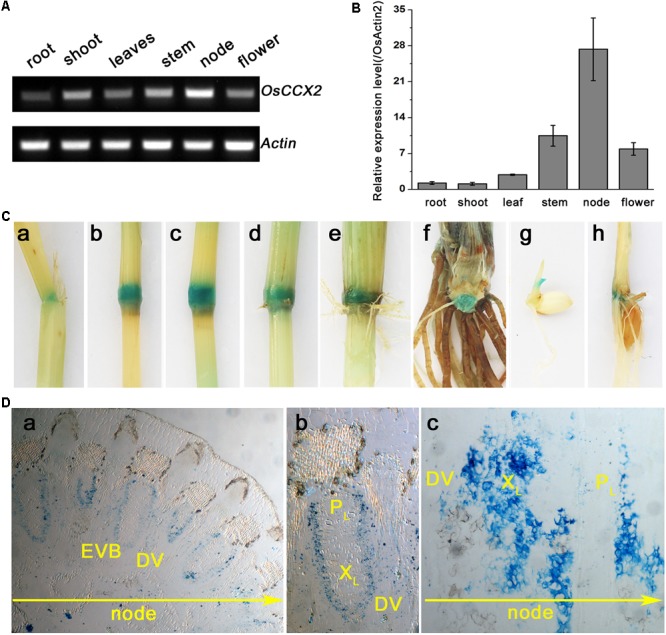
Tissue expression pattern analyses of *OsCCX2*. **(A)** The semi-quantitative-RT-PCR analysis of *OsCCX2* transcript levels. Data are representative values of three independent experiments. **(B)** The q-PCR analysis of *OsCCX2* transcript levels in diverse tissues. Data are average values of three independent experiments and are presented as mean ± *SD*. **(C)** Histochemical GUS staining of *OsCCX2 pro*:GUS plants. (a–f) The maturation plant grown on soil. (a) The leaf sheath. (b) Node I. (c) Node II. (d) Node III. (e) Node IV. (f) Unelongated basal stem. (g) Four-day-old seedling grown on MS agar plates. (h) Two-week-old seedling grown in hydroponics. **(D)** Paraffin section of Node I. The GUS signal is shown in blue. (a) Cross slice of Node I. (b) Enlarged image of an EVB and a DVB in (a). (c) Longitudinal slice of Node I. Bar = 200 μm.

To confirm the preferential expression of *OsCCX2* in node tissues, we first analyzed the expression pattern of *OsCCX2* by semi-quantitative RT-PCR approach and detected obvious DNA band in the node tissue, but weakly in other tissues (**Figure 1A**). Subsequently, q-PCR was performed to quantify the expression of *OsCCX2*, similar results were obtained in that the node tissue has the highest expression, while flower and culm (excluding nodes) tissues have comparatively higher expression, but root and shoot of the seedlings just have basal expression (**Figure 1B**). To further clarify the expression pattern of OsCCX2 in detail, we generated the *OsCCX2* promoter driven GUS transgenic rice. Strong GUS signal was detected only in node tissues (**Figure 1C**), while weak GUS signal was detected in internode tissues, consistent with the results by quantitative PCR. Paraffin section analysis revealed that GUS activity is mainly localized in the parenchyma cells of the vascular tissues including enlarged vascular bundles (EVBs) and diffuse vascular bundles (DVBs) in the node (**Figure 1D** and Supplementary Figure S1). As the EVBs and DVBs play important roles in cation upward transport to the internode and leaves, the preferential expression of *OsCCX2* at the node of the culm suggests its linkage to cation accumulation in grains.

### Disruption of *OsCCX2* Led to Decreased Cd Accumulation in the Grains of the *ccx2* Mutants

**FIGURE 2:**
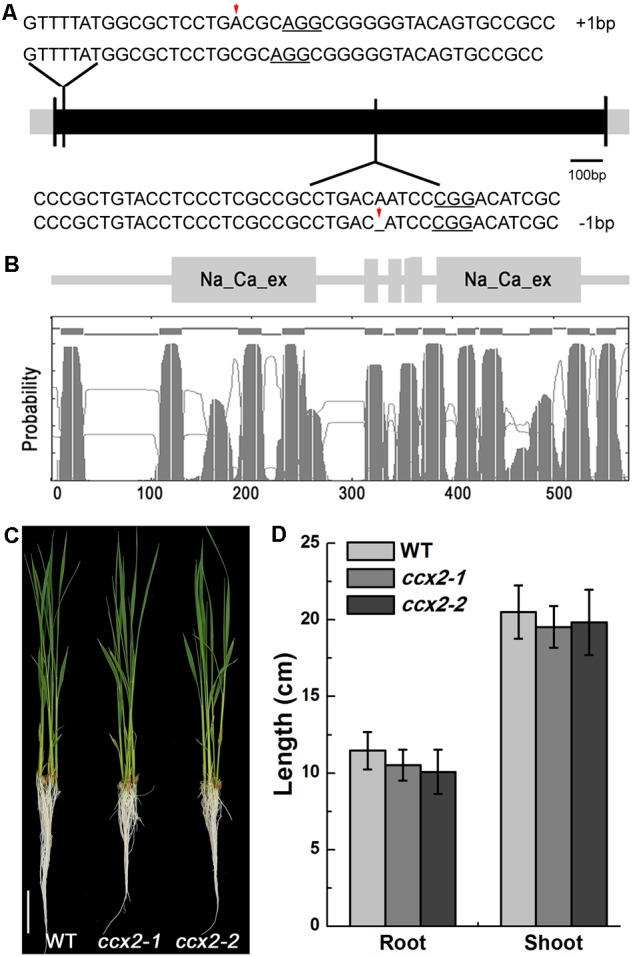
OsCCX2 structure analysis and *ccx2* mutant generation. **(A)** Targeted mutagenesis of OsCCX2 gene by CRISPR-Cas9. Two independent gene edition sites were designed (NGG motifs underlined). Sequences of the mutant alleles are aligned to the genome sequence of wild type, and two homozygous mutant lines (*ccx2-1* and *ccx2-2*) were obtained with 1 bp insertion and 1 bp deletion separately (shown by red arrows). **(B)** Schematic topology diagrams of OsCCX2. OsCCX2 protein with 12 transmembrane domains and two Na/Ca exchanger domains. **(C,D)** Seedlings of two homozygous mutant lines (*ccx2-1* and *ccx2-2*). The germinated seeds were grown in hydroponic solution for 4 weeks **(C)**, and the lengths of roots and shoots of the wild type and mutant plants were measured (*n* = 20 for each data point) **(D)**. Data are average values of three independent experiments and are presented as mean ± *SD*. Bar = 3 cm **(C)**.

To dissect the function of OsCCX2, two independent gene knockout mutants were obtained by using the CRISPR/Cas9 gene editing technology. First, two sgRNAs were designed that could target the editing sites based on the sequence of the *OsCCX2*, where one was located at 17 bp of exon from ATG and another was located at 986 bp of exon (**Figure 2A**). Then the recombinant vectors that contained sgRNA and Cas9 were transformed into *Nipponbare*, a cultivar of *Oryza sativa* japonica by *Agrobacterium*-mediated transformation method. The mutants were screened by hygromycin from the regenerated seedlings of the transformed plants, and were determined by sequencing using specific primers (Supplementary Table S1). A total of 31 transgenic lines were confirmed being edited successfully with nucleotide(s) deletion or insertion in the targeting site 1 and 21 lines in site 2, respectively (Supplementary Table S2), from which two independent homozygous lines, *ccx2-1* and *ccx2-2*, were chosen for further analysis, where the *OsCCX2* mutation occurred with one nucleotide insertion at 12 bp from ATG of site 1 in *ccx2-1* and one nucleotide deletion at 986 bp from ATG of site 2 in *ccx2-2*, resulting in frameshift mutation and precocious translation termination (**Figure 2A**). Subsequently, the growth stature between the *ccx2* mutants and wild type plants was observed. The mutants showed a little bit shorter both in roots and shoots of the seedlings than the wild type plants, but without significant difference (**Figures 2C,D**).

**FIGURE 3:**
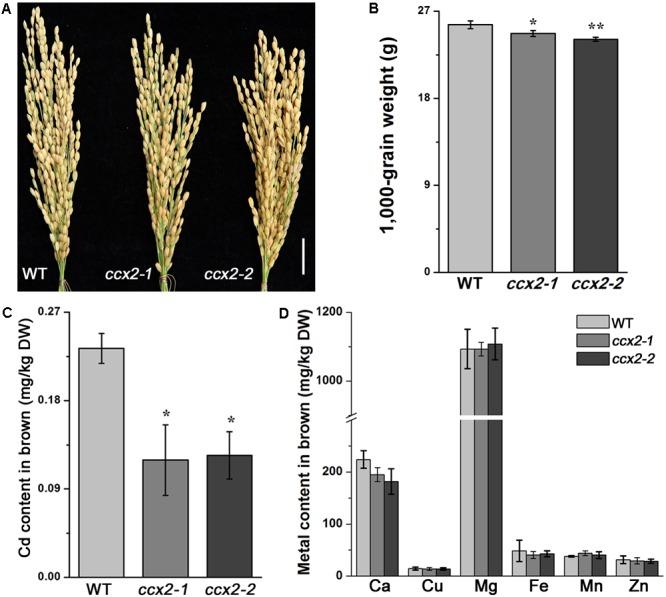
Cadmium content in different organs of rice. *Nipponbare* wild type rice and the *ccx2-1* and *ccx2-2* mutant lines were grown in Cd-containing paddy soil (3.9 mg/kg) till ripening. The Cd and other metal content in grains were detected. Data for Cd content and 1000-grain weight are average values of three independent experiments and are presented as mean ± *SD*. Significant differences are labeled “^∗^” (*p* < 0.05) or “^∗∗^” (*p* < 0.01). **(A)** The morphology of rice panicles. Bar = 2 cm. **(B)** The 1000-grain weight. **(C)** The content of Cd in brown rice of the mutant and wild type rice. **(D)** The content of other metals in brown.

To explore how *OsCCX2* is involved in Cd accumulation in rice, we compared the Cd content between the mutants and the wild type plants. First, the mutants (*ccx2-1* and *ccx2-2*) and wild type plants were grown in the paddy soil (Cd, 3.89 mg/kg) till grain ripening. The setting percentage showed no different between mutants and wild type, but the weight of 1000 grains of *ccx2* mutants (24.4 and 24.0 g for *ccx2-1* and *ccx2-2*, respectively) was reduced by 4.7 and 6.3%, respectively, compared with that of wild type (25.6 g for the wild type), where significant differences displayed between *ccx2* mutants and the wild type (*p* < 0.05; **Figures 3A,B**). Grains of different lines were then harvested and the Cd content in these grains was quantified by GFAAS. The Cd content in browns of the two mutants is roughly the same, up to 0.12 mg/kg DW, while the Cd content in browns of wild type *Nipponbare* is 0.23 mg/kg DW, indicating that disruption of *OsCCX2* gene dramatically decreased Cd accumulation in grains (**Figure 3C**). We further detected some other divalent cations such as Ca, Fe, Cu, Mg, and Zn between the mutant and wild type browns, and found no obvious difference in content except for Ca (**Figure 3D**), suggesting that OsCCX2 might mediate Ca transport, in consistence with the result in yeast (Yadav et al., 2015). These results showed that OsCCX2 is involved in grain Cd accumulation when the rice is grown in Cd polluted paddy field.

### OsCCX2 Participates in Root-to-Shoot Translocation of Cd

**FIGURE 4:**
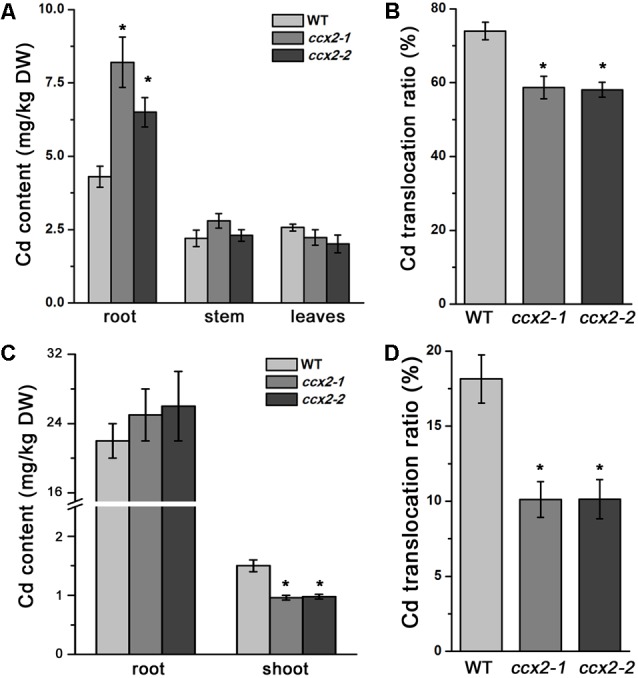
Root-to-shoot translocation of Cd in plant. The plants were grown in paddy soil till booting, then Cd content of root, culm, and leaves was measured **(A)**, and root-to-shoot translocation ratio of Cd was calculated **(B)**. **(C,D)** 10-day-old seedlings were transplanted to Cd-containing (0.1 μM) hydroponic solution for 7 days. Cd content in root and shoot was measured **(C)**, and Cd translocation ratio of shoot-to-root was calculated **(D)**. Data are average values of three independent experiments and are presented as mean ±*SD*. Asterisks above the bars indicate significant difference (^∗^*p* < 0.05) compared with the WT rice.

Besides Cd content in browns, we also detected Cd in roots, culms, and leaves. The Cd content of roots was significantly higher in *ccx2* mutant than in wild type. However, the Cd content of culms and leaves was a little bit lower in *ccx2* mutant than in wild type, but showed no significant difference, suggesting that root-to-shoot translocation ratio was impacted when *OsCCX2* was disrupted (**Figure 4A**).

We subsequently compared the translocation ratio of Cd by measuring Cd accumulation in the mature organs. The root-to-shoot translocation ratio was 73.9% in wild type plants. However, the root–to-shoot translocation ratio reduced to 58.6 and 58%, respectively, in *ccx2-1* and *ccx2-2* mutants, significantly lower than that in wild type rice (**Figure 4B**), indicating that OsCCX2 is involved in the root-to-shoot Cd translocation.

We further analyzed Cd accumulation characteristic in the seedlings. The germinated seeds were grown by hydroponic culture for 10 days, and then transferred to hydroponic solution containing different concentrations of Cd to grow continuously for another 7 days, and the Cd content of the root and shoot was determined. When grown in 0.1 μM external environmental Cd, the average Cd content in the root of *ccx2* mutants (25.50 mg/kg DW) is slightly higher than that of the wild type (22.0 mg/kg DW); however, the average Cd content in the shoot of *ccx2* mutants (0.97 mg/kg DW) is significantly lower than that of the wild type (1.50 mg/kg DW) respectively (**Figure 4C**). The root-to-shoot translocation ratio of Cd in *ccx2* mutants (10.1%) is obviously lower than that in the wild type (18.1%; **Figure 4D**). When grown in 5 μM external environmental Cd, the average Cd content in the root of *ccx2* mutants (236.6 mg/kg DW) is significantly higher than that of the wild type (176.95 mg/kg DW), while the average Cd content in the shoot of *ccx2* mutants (5.98 mg/kg DW) is significantly lower than that of the wild type (8.99 mg/kg DW) respectively (Supplementary Figure S2A). The calculated root-to-shoot translocation ratio of *ccx2* mutants (average, 5.0%) is significantly lower than that of the wild type (7.75%; *p* < 0.01; Supplementary Figure S2B).

We also observed the Cd accumulation in the roots with Cd fluorescent dye when they were grown in the 5 μM external environmental Cd. The roots of the *ccx2* mutant and the wild type were stained by Leadmium^TM^ Green AM fluorescent dye and the fluorescent signals were detected under confocal microscopy. The Cd content was evaluated by Image J analysis based on fluorescent intensity (Supplementary Figure S2C). The roots of *ccx2* mutant showed higher fluorescent intensity than those of the wild type in both root apical meristem zone and elongation zone, implicating that the Cd content in the roots of *ccx2* mutant is higher than that in the wild type. All these data support that *OsCCX2* participates in root-to-shoot translocation of Cd.

### OsCCX2 Is Involved in Cd Distribution in Shoot

**FIGURE 5:**
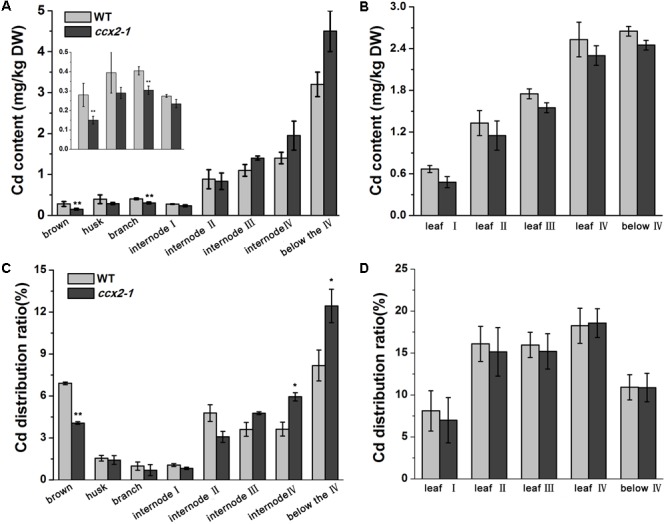
Cd distribution in shoot tissues of rice. The *ccx2-1* mutant line and the wild type control were grown in Cd-containing paddy soil (3.9 mg/kg) till ripening. Different tissues of the shoot were separated, including brown, husk, branch, each internode, and each leaf. The Cd content was determined and the distribution ratios were calculated. Three independent experiments were performed, and values represent means ±*SD*. Asterisks above the bars indicate significant difference (^∗^*p* < 0.05). **(A,B)** The Cd content in the shoot tissues, including brown, husk, branch, each internode, and each leaf (labeled I to “below” from top to bottom). **(C,D)** The Cd distribution ratios of the shoot tissues.

**FIGURE 6:**
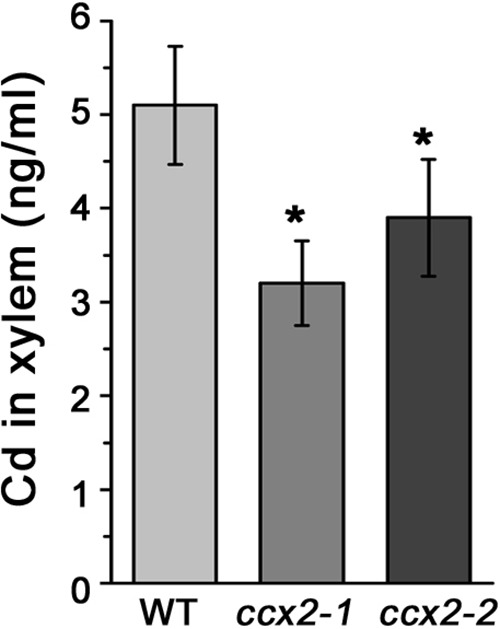
Cd content in xylem sap. The *ccx2-1* and *ccx2-2* mutant lines and the wild type control were grown in Cd-containing paddy soil (1.2 mg/kg) till grain-filling initiation, and then the xylem sap was collected from a cut in the middle of the uppermost internode. Data are average values of three independent experiments and are presented as mean ±*SD*. Asterisks above the bars indicate significant differences (^∗^*p* < 0.05) compared with the WT rice.

To further understand Cd distribution in the shoot, we measured the Cd content of different parts of the shoot, including grains, branch, each internode, and each leaf at different levels. After heading, *Nipponbare* shoot usually generate four visible nodes, internodes, and functional leaves, labeled number I–IV according to its position from top to bottom (Yamaji and Ma, 2014). The result showed that the Cd content presented a decreased tendency from bottom to top of the shoot in both the wild type and *ccx2* mutant plants. Furthermore, the Cd content of the internodes and leaves showed a similar tendency from position IV to position I. Interestingly, although the total Cd accumulation is lower in the shoot of the *ccx2* mutant than that in the wild type, the *ccx2* mutant displayed higher Cd content and proportion at the lower part of the culm than the wild type, and gradually decreased its Cd accumulation from bottom to top, till finally fell into lower Cd proportion and content at the upper part of the culm. Compared to the wild type, the *ccx2* mutant accumulated higher Cd in basal internodes III, IV, and below IV, but accumulated less Cd in leaves and in the upper parts of the culm including internodes I and II, as well as branch and grain (**Figure 5**). Besides, sap analysis showed that the Cd content in xylem sap of the *ccx2* upper internode is lower than wild type at the grain filling stage, consistent with the lower Cd content in panicle tissues (**Figure 6**). And the Ca and Cd contents in the first leaf in mutant were lower than wild type as well when they were grown in different Cd content soil (Supplementary Figure S3). To sum up, disruption of *OsCCX2* impacts the distribution of Cd in above-ground parts.

### OsCCX2 Might Function as an Efflux Transporter

**FIGURE 7:**
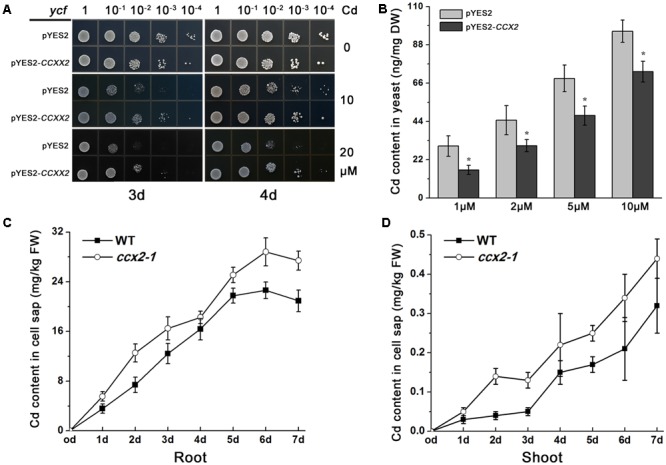
Cd analysis in *OsCCX2*-expressing yeast cells and in cell sap of the *ccx2* plant. **(A)** Cadmium tolerance of yeast cells expressing *OsCCX2*. Yeast liquid cultures were grown and adjusted to an optical density of 0.5 at OD600, and 5 μl aliquots of serial dilutions (10^-1^,10^-2^, 10^-3^, and 10^-4^) were plated onto SD-Ura medium without CdCl_2_ (the upper two rows), or containing certain concentrations of CdCl_2_ (10 μM for the middle two rows and 20 μM for the bottom two rows). The plates were further incubated at 30°C for 3 days (left panels) and 4 days (right panels), respectively. The *ycf1* mutants were transformed pYES2 empty vector or pYES2-*OsCCX2*. Data are representative values of three independent experiments. **(B)** Cd content of transformants grown on CdCl_2_-containing media. The pYES2-*OsCCX2* and pYES2 empty vectors were transformed into wild type yeast BY4741, respectively. The transformants were grown on SD liquid cultures for 24 h (containing 1, 2, 5, and 10 μM CdCl_2_, respectively), and the Cd content was determined. Data are average values of three independent experiments and are presented as mean ± *SD*. **(C,D)** Two-week-old seedlings were transplanted to 5 μM Cd-containing hydroponic solution, and the shoots and roots were collected at 1, 2, 3, 4, 5, 6, and 7 days. The shoot and the root were homogenized and subcellular fractions were separated by using differential centrifugation techniques. Data are average values of three independent experiments and are presented as mean ±*SD*.

The yeast cadmium factor 1 (YCF1) is a tonoplast membrane transporter, mediating both GSH and (GSH)_2_-Cd transport into the tonoplast in yeast (Li et al., 1996), and *YCF1* deletion causes *ycf1* mutant cells hypersensitive to Cd. When *ycf1* mutants were transformed with pYES2 empty vector or pYES2-*OsCCX2* respectively, the yeast liquid cultures were plated onto SG-Ura medium with concentrations of CdCl_2_ (0, 10, and 20 μM). The plates were further incubated at 30°C for 3 and 4 days, respectively (**Figure 7A**). The results showed that *ycf1* mutant cells transformed with pYES2-*OsCCX2* display stronger tolerance to Cd than the control. To determine whether the enhanced tolerance to Cd is due to less Cd accumulation or Cd sequestered into the vacuole of the *OsCCX2*-expressing yeast cells, the pYES2-*OsCCX2* were transformed into yeast BY4741, then the transformants were incubated on SG-Ura liquid medium for 24 h (containing 1, 2, 5, and 10 μM CdCl_2_, respectively), and the yeasts were collected to determine the Cd content. As shown in **Figure 7B**, the *OsCCX2*-expressing yeast cells accumulate significantly less Cd, compared with the controls, suggesting that OsCCX2 might be plasma membrane – localized, and has efflux transport activity.

To clarify whether mutation of OsCCX2 affects intracelluar Cd content in plant, the cell sap Cd content was detected referring to the suggested method (Weigel and Jager, 1980). Briefly, 7-day-old seedlings were transplanted to Cd-containing hydroponic solution to grow for another 1, 2, 3, 4, 5, 6, and 7 days respectively, and then the shoot and root tissues were collected. The soluble fraction of each sample was extracted by using centrifugation technique. The results showed that the Cd concentration in the cell sap was higher both in root and shoot of the *ccx2* mutants than that in the wild type plants (**Figures 7C,D**), indicating that Cd outward transport from cell sap was blocked when OsCCX2 was disrupted. These suggest that OsCCX2 might function as an efflux transporter in the plasma membrane.

### OsCCX2 Is Localized in Plasma Membrane

**FIGURE 8:**
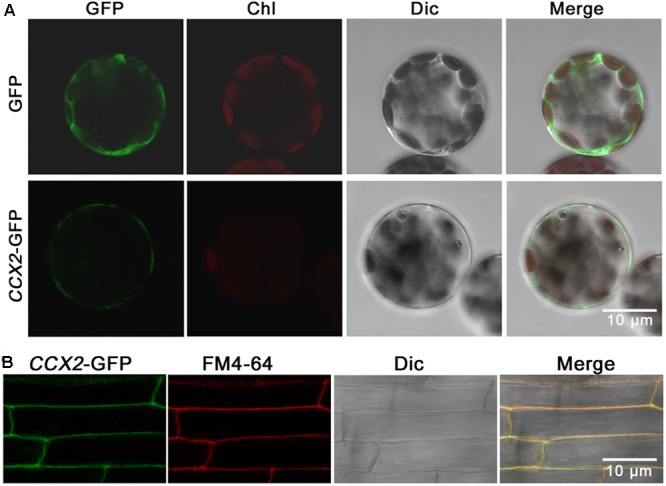
Subcellular localization of *OsCCX2*. The *OsCCX2* coding sequence was fused to the N terminus of the GFP coding region in the pCambia2300 and pCambia1302 vector separately, and then transformed into *Arabidopsis* mesophyll protoplasts and wild type *Nipponbare*, respectively. The empty vector pCambia2300-transformed protoplasts were used as the control. The fluorescent signals were imaged by using an LSM710 confocal laser scanning microscope. Bars = 10 μm. **(A)** OsCCX2-GFP signal in *Arabidopsis* mesophyll protoplasts. The green signals were from GFP, and the red signals were from chloroplasts. **(B)** OsCCX2-GFP signal in root tip cells of *Nipponbare* rice. The green fluorescence came from GFP signals, and the red signals were from plasma membrane-specific fluorescent dye FM4-64. The yellow signals were merges of the green and red signals.

To determine the subcellular localization of OsCCX2, a transient expression vector containing an OsCCX2-GFP cassette driven by CaM 35S promoter was constructed and introduced into *Arabidopsis* mesophyll protoplasts. The GFP fluorescent signals in protoplasts were detected in the plasma membrane, suggesting that OsCCX2 is localized in the plasma membrane (**Figure 8A**).

To confirm the subcellular localization, the *OsCCX2* coding sequence without stop codon was ligated into a binary vector pCambia1302 to form an OsCCX2-GFP cassette and transformed into rice (*Nipponbare*) to generate the transgenic plants. The GFP signals in the root tissues were detected under confocal microscopy. The GFP signals were well overlapped with the red fluorescent signals produced by the plasma membrane-specific dye FM4-64, indicating that OsCCX2 is located in plasma membrane (**Figure 8B**).

## Discussion

The *OsCCX2* is composed of 576 amino acid residues and possesses 12 transmembrane domains and two Na/Ca exchanger domains (**Figure 2B**). Our results confirmed that *OsCCX2* plays an important role in Cd transport by impacting Cd root-to-shoot translocation and Cd distribution at the above-ground tissues. Mutation of *OsCCX2* resulted in low Cd accumulation in the grains and thus has potential application value to generate low Cd accumulation rice resource for Cd contaminated area. As Cd is a toxic, non-essential cation for plants, plants are unlikely to evolve specific transporter to uptake and transport Cd. Several transporters responsible for transport some bivalent cations, such as Zn, Mn, and Fe, were identified involving in this process. OsNRAMP5 functions as a Mn transporter to uptake Mn and Cd in the root from outside environment (Ishimaru et al., 2012b; Sasaki et al., 2012; Takahashi et al., 2014; Yang et al., 2014; Tang et al., 2017). Ferrous Fe transporters, OsIRT1, OsIRT2, and NRAMP1, have been shown to participate in root Cd uptake and transport within plants (Nakanishi et al., 2006; Lee and An, 2009; Takahashi et al., 2011a,b). *OsHMA2* is mainly expressed in root, mediating the root-to-shoot translocation of Zn/Cd (Satoh-Nagasawa et al., 2012; Takahashi et al., 2012a; Yamaji et al., 2013). *OsHMA2* lose-of-function mutant showed marked Cd reduction in grain and shoots. However, the mutant plant height and grain yield were impaired, possibly due to Zn deficiency in the above-ground tissues (Yamaji et al., 2013). OsHMA2 is also expressed at nodes of the culm at the reproductive growth stage, mediating intervascular mobilization of Zn and Cd (Takahashi et al., 2012b; Yamaji et al., 2013). *OsHMA3* is responsible for Cd sequestration into tonoplasts in root cells (Miyadate et al., 2011; Yan et al., 2016). Furthermore, enhanced expression of OsHMA3 promoted Zn and Cd transport into the tonoplast and increased the Zn/Cd accumulation in the roots of the overexpression lines (Sasaki et al., 2014). Our work reports for the first time a putative a Ca transporter that is involved in Cd transport. OsCCX2 might function as an efflux transporter, responsible for Cd/Ca loading to xylem vessels from surrounding parenchyma cells.

Grain Cd accumulation in rice comes from two pathways. One is Cd remobilization from vegetative organs especially from leaves via phloem, and another is direct transport from root-absorbed Cd via xylem at the grain-filling stage. A timing analysis of grain Cd accumulation has been performed by Rodda et al. (2011). They found that both pathways contribute to grain Cd accumulation, of which about 60% of grain Cd was remobilized from vegetative tissue pre-deposited before flowering and 40% of grain Cd came from novel Cd uptake during grain-filling stage (Rodda et al., 2011). The node has complex vascular bundle net, linking with internodes and sheath, where crosstalk takes place among EVBs and DVBs, or even between xylem and phloem, and thus is crucial for substance transport and interchange, and finally affecting the distribution of the substance including Cd, where cation transporters at the nodes play important role in cation distribution to the above-ground tissues including grain. Up to now, only three node-expressed Cd transporters have been identified, and they seem to have diverse responsibilities. Among them, OsHMA2 is expressed at the phloem, as an influx transporter, responsible for Cd transfer from phloem to xylem, while OsLCT1 is also expressed in phloem of the node, but functions as efflux transporter, involved in loading Cd into phloem sieve tube. These two transporters may participate in Cd remobilization from vegetative organs to grains. OsCCX2 is expressed in xylem, might function in loading Cd into xylem vessel, and mediate direct root-derived Cd transport to grain. As knockout of a single gene can lead to low Cd accumulation in grain, it is promising to obtain novel low Cd rice germplasm resource by generating double mutant or even triple mutant of these genes. The role of node in Cd distribution will be better understood when the mechanisms of more node-expressed Cd transporters are revealed in the future.

The subcellular localization information is very important to understand the function of a protein. By expressing OsCCX2-GFP in rice and *Arabidopsis* mesophyll protoplast, we localized OsCCX2 to the plasma membrane, consistent with the result in rice leaf sheath cells by Kuramata et al. (2009). That seems to make sense to explain the OsCCX2-expressing yeast and *Arabidopsis* cells accumulated less Cd in comparison with the wild type (Kuramata et al., 2009). However, there was report showing that OsCCX2 was targeted to tonoplast rather than the plasma membrane in a tobacco epidermal cell by Yadav et al. (2015). The reason for this discrepancy is unclear and needs to be clarified further. Possibly, OsCCX2 targets to both sites. A lot of transporters have been shown to be multiple-localized. For example, the auxin transporter PIN6 is localized to both plasma membrane and endoplamic reticulum (Simon et al., 2016). Also, environmental factors could lead to some protein relocating. A P-type H^+^-ATPase, AHA2, is a plasma membrane transporter, and could traffic into intracellular compartments in response to environmental and developmental changes such as light, pH, or even age-dependent cell types (Haruta et al., 2018). *OsCCX2* is upregulated under drought and salt stress and downregulated under Ca deficiency (Yadav et al., 2015). It is possible for OsCCX2 to locate both in plasma membrane and tonoplast to mediate Ca homeostasis and respond to environmental stress.

OsCCX2 was identified as a functional K^+^-independent Ca transporter in yeast (Yadav et al., 2015). We also observed reduced Ca accumulations in grains and flag leaf of the *ccx2* mutants, providing evidence for Ca transport activity of OsCCX2 (**Figure 3D** and Supplementary Figure S3A). In a word, we proposed that a putative CCX, OsCCX2, transports Cd and Ca in the nodes and mediates grain Cd accumulation.

## Author Contributions

XH, DL, LC, and LT designed the research. XH, MZ, JW, ZZ, ZX, JD, YY, and LT performed the research. XH, DL, LC, and LT analyzed the data and wrote the paper.

## Conflict of Interest Statement

The authors declare that the research was conducted in the absence of any commercial or financial relationships that could be construed as a potential conflict of interest.

## References

[B1] Buha A. Wallace D. Matovic V. Schweitzer A. Oluic B. Micic D. (2017). Cadmium exposure as a putative risk factor for the development of pancreatic cancer: three different lines of evidence. *Biomed. Res. Int.* 8:8. 10.1155/2017/1981837 29349066 PMC5733953

[B2] Cai X. Lytton J. (2004). The cation/Ca(2+) exchanger superfamily: phylogenetic analysis and structural implications. *Mol. Biol. Evol.* 21 1692–1703. 10.1093/molbev/msh177 15163769

[B3] Clemens S. Aarts M. G. M. Thomine S. Verbruggen N. (2013). Plant science: the key to preventing slow cadmium poisoning. *Trends Plant Sci.* 18 92–99. 10.1016/j.tplants.2012.08.003 22981394

[B4] Emery L. Whelan S. Hirschi K. D. Pittman J. K. (2012). Protein phylogenetic analysis of Ca2+/cation antiporters and insights into their evolution in plants. *Front. Plant Sci.* 3:1. 10.3389/fpls.2012.00001 22645563 PMC3355786

[B5] Feki-Tounsi M. Hamza-Chaffai A. (2014). Cadmium as a possible cause of bladder cancer: a review of accumulated evidence. *Environ. Sci. Pollut. Res.* 21 10561–10573. 10.1007/s11356-014-2970-0 24894749

[B6] Garg R. Tyagi A. K. Jain M. (2012). Microarray analysis reveals overlapping and specific transcriptional responses to different plant hormones in rice. *Plant Signal. Behav.* 7 951–956. 10.4161/psb.20910 22827941 PMC3474693

[B7] Goyer R. A. Liu J. Waalkes M. P. (2004). Cadmium and cancer of prostate and testis. *Biometals* 17 555–558. 10.1023/b:biom.0000045738.59708.20 15688863

[B8] Guthrie C. Fink R. G. (2002). Guide to yeast genetics and molecular and cell biology. *Methods Enzymol.* 350 18–19.

[B9] Haruta M. Tan L. X. Bushey D. B. Swanson S. J. Sussman M. R. (2018). Environmental and genetic factors regulating localization of the plant plasma membrane H+-ATPase. *Plant Physiol.* 176 364–377. 10.1104/pp.17.01126 29042459 PMC5761788

[B10] Horiguchi H. Teranishi H. Niiya K. Aoshima K. Katoh T. Sakuragawa N. (1994). Hypoproduction of erythropoietin contributes to anemia in chronic cadmium intoxication: clinical study on Itai-itai disease in Japan. *Arch. Toxicol.* 68 632–636. 10.1007/bf03208342 7857202

[B11] Huff T. B. Tong L. Zhao Y. Hansen M. N. Cheng J.-X. Wei A. (2007). Hyperthermic effects of gold nanorods on tumor cells. *Nanomedicine* 2 125–132. 10.2217/17435889.2.1.125 17716198 PMC2597406

[B12] Il’yasova D. Schwartz G. G. (2005). Cadmium and renal cancer. *Toxicol. Appl. Pharmacol.* 207 179–186. 10.1016/j.taap.2004.12.005 16102569

[B13] Ishimaru Y. Bashir K. Nakanishi H. Nishizawa N. K. (2012a). OsNRAMP5, a major player for constitutive iron and manganese uptake in rice. *Plant Signal. Behav.* 7 763–766. 10.4161/psb.20510 22751306 PMC3583959

[B14] Ishimaru Y. Takahashi R. Bashir K. Shimo H. Senoura T. Sugimoto K. (2012b). Characterizing the role of rice NRAMP5 in manganese, iron and cadmium transport. *Sci. Rep.* 2:286. 10.1038/srep00286 22368778 PMC3285952

[B15] Jefferson R. A. Kavanagh T. A. Bevan M. W. (1987). GUS fusions: beta-glucuronidase as a sensitive and versatile gene fusion marker in higher plants. *EMBO J.* 6 3901–3907. 3327686 10.1002/j.1460-2075.1987.tb02730.x PMC553867

[B16] Jin Y. H. Clark A. B. Slebos R. J. C. Al-Refai H. Taylor J. A. Kunkel T. A. (2003). Cadmium is a mutagen that acts by inhibiting mismatch repair. *Nat. Genet.* 34 326–329. 10.1038/ng1172 12796780 PMC2662193

[B17] Kobayashi T. Itai R. N. Nishizawa N. K. (2014). Iron deficiency responses in rice roots. *Rice* 7:27. 10.1186/s12284-014-0027-0 26224556 PMC4884003

[B18] Kuramata M. Masuya S. Takahashi Y. Kitagawa E. Inoue C. Ishikawa S. (2009). Novel cysteine-rich peptides from *Digitaria ciliaris* and *Oryza sativa* enhance tolerance to cadmium by limiting its cellular accumulation. *Plant Cell Physiol.* 50 106–117. 10.1093/pcp/pcn175 19017626

[B19] Lancilli C. Giacomini B. Lucchini G. Davidian J.-C. Cocucci M. Sacchi G. A. (2014). Cadmium exposure and sulfate limitation reveal differences in the transcriptional control of three sulfate transporter (Sultr1;2) genes in *Brassica juncea*. *BMC Plant Biol.* 14:132. 10.1186/1471-2229-14-132 24884748 PMC4049391

[B20] Lee S. An G. (2009). Over-expression of OsIRT1 leads to increased iron and zinc accumulations in rice. *Plant Cell Environ.* 32 408–416. 10.1111/j.1365-3040.2009.01935.x 19183299

[B21] Li J. Huang Y. Tan H. Yang X. Tian L. Luan S. (2015). An endoplasmic reticulum magnesium transporter is essential for pollen development in Arabidopsis. *Plant Sci.* 231 212–220. 10.1016/j.plantsci.2014.12.008 25576006

[B22] Li Z. Wang X. Chen J. Gao J. Zhou X. Kuai B. (2016). CCX1, a putative Cation/Ca2+ exchanger, participates in regulation of reactive oxygen species homeostasis and leaf senescence. *Plant Cell Physiol.* 57 2611–2619. 10.1093/pcp/pcw175 27986916

[B23] Li Z. S. Szczypka M. Lu Y. P. Thiele D. J. Rea P. A. (1996). The yeast cadmium factor protein (YCF1) is a vacuolar glutathione S-conjugate pump. *J. Biol. Chem.* 271 6509–6517. 10.1074/jbc.271.11.6509 8626454

[B24] Lutzen A. Liberti S. E. Rasmussen L. J. (2004). Cadmium inhibits human DNA mismatch repair in vivo. *Biochem. Biophys. Res. Commun.* 321 21–25. 10.1016/j.bbrc.2004.06.102 15358209

[B25] Matovic V. Buha A. Bulat Z. Dukic-Cosic D. (2011). Cadmium toxicity revisited: focus on oxidative stress induction and interactions with zinc and magnesium. *Arh. Hig. Rada Toksikol.* 62 65–76. 10.2478/10004-1254-62-2011-2075 21421535

[B26] Matovic V. Buha A. Dukic-Cosic D. Bulat Z. (2015). Insight into the oxidative stress induced by lead and/or cadmium in blood, liver and kidneys. *Food Chem. Toxicol.* 78 130–140. 10.1016/jict.2015.02.011 25681546

[B27] Matsuda T. Kuramata M. Takahashi Y. Kitagawa E. Youssefian S. Kusano T. (2009). A novel plant cysteine-rich peptide family conferring cadmium tolerance to yeast and plants. *Plant Signal. Behav.* 4 419–421. 19816106 10.4161/psb.4.5.8272 PMC2676753

[B28] Miao J. Guo D. Zhang J. Huang Q. Qin G. Zhang X. (2013). Targeted mutagenesis in rice using CRISPR-Cas system. *Cell Res.* 23 1233–1236. 10.1038/cr.2013.123 23999856 PMC3790239

[B29] Misra U. K. Gawdi G. Pizzo S. V. (2003). Induction of mitogenic signalling in the 1LN prostate cell line on exposure to submicromolar concentrations of cadmium+. *Cell. Signal.* 15 1059–1070. 10.1016/s0898-6568(03)00117-7 14499349

[B30] Miyadate H. Adachi S. Hiraizumi A. Tezuka K. Nakazawa N. Kawamoto T. (2011). OsHMA3, a P-1B-type of ATPase affects root-to-shoot cadmium translocation in rice by mediating efflux into vacuoles. *New Phytol.* 189 190–199. 10.1111/j.1469-8137.2010.03459.x 20840506

[B31] Moon S. Kim S.-R. Zhao G. Yi J. Yoo Y. Jin P. (2013). Rice GLYCOSYLTRANSFERASE1 encodes a glycosyltransferase essential for pollen wall formation. *Plant Physiol.* 161 663–675. 10.1104/pp.112.210948 23263792 PMC3561011

[B32] Morris J. Tian H. Park S. Sreevidya C. S. Ward J. M. Hirschi K. D. (2008). AtCCX3 is an Arabidopsis endomembrane H+-dependent K+ transporter. *Plant Physiol.* 148 1474–1486. 10.1104/pp.108.118810 18775974 PMC2577254

[B33] Nakanishi H. Ogawa I. Ishimaru Y. Mori S. Nishizawa N. K. (2006). Iron deficiency enhances cadmium uptake and translocation mediated by the Fe2+ transporters OsIRT1 and OsIRT2 in rice. *Soil Sci. Plant Nutr.* 52 464–469. 10.1111/j.1747-0765.2006.00055.x

[B34] Rapisarda V. Miozzi E. Loreto C. Matera S. Fenga C. Avola R. (2018). Cadmium exposure and prostate cancer: insights, mechanisms and perspectives. *Front. Biosci. (Landmark Ed.)* 23:1687–1700. 29293457 10.2741/4667

[B35] Rodda M. S. Li G. Reid R. J. (2011). The timing of grain Cd accumulation in rice plants: the relative importance of remobilisation within the plant and root Cd uptake post-flowering. *Plant Soil* 347 105–114. 10.1007/s11104-011-0829-4

[B36] Sasaki A. Yamaji N. Ma J. F. (2014). Overexpression of OsHMA3 enhances Cd tolerance and expression of Zn transporter genes in rice. *J. Exp. Bot.* 65 6013–6021. 10.1093/jxb/eru340 25151617 PMC4203134

[B37] Sasaki A. Yamaji N. Mitani-Ueno N. Kashino M. Ma J. F. (2015). A node-localized transporter OsZIP3 is responsible for the preferential distribution of Zn to developing tissues in rice. *Plant J.* 84 374–384. 10.1111/tpj.13005 26332571

[B38] Sasaki A. Yamaji N. Yokosho K. Ma J. F. (2012). Nramp5 is a major transporter responsible for manganese and cadmium uptake in rice. *Plant Cell* 24 2155–2167. 10.1105/tpc.112.096925 22589467 PMC3442593

[B39] Satoh-Nagasawa N. Mori M. Nakazawa N. Kawamoto T. Nagato Y. Sakurai K. (2012). Mutations in Rice (*Oryza sativa*) heavy metal ATPase 2 (OsHMA2) restrict the translocation of zinc and cadmium. *Plant Cell Physiol.* 53 213–224. 10.1093/pcp/pcr166 22123790

[B40] Simon S. Skupa P. Viaene T. Zwiewka M. Tejos R. Klima P. (2016). PIN6 auxin transporter at endoplasmic reticulum and plasma membrane mediates auxin homeostasis and organogenesis in Arabidopsis. *New Phytol.* 211 65–74. 10.1111/nph.14019 27240710

[B41] Singh A. Kanwar P. Yadav A. K. Mishra M. Jha S. K. Baranwal V. (2014). Genome-wide expressional and functional analysis of calcium transport elements during abiotic stress and development in rice. *FEBS J.* 281 894–915. 10.1111/febs.12656 24286292

[B42] Song J. K. Luo H. Yin X. H. Huang G. L. Luo S. Y. Lin D. R. (2015). Association between cadmium exposure and renal cancer risk: a meta-analysis of observational studies. *Sci. Rep.* 5:17976. 10.1038/srep17976 26656678 PMC4675972

[B43] Su Y. Liu J. Lu Z. Wang X. Zhang Z. Shi G. (2014). Effects of iron deficiency on subcellular distribution and chemical forms of cadmium in peanut roots in relation to its translocation. *Environ. Exp. Bot.* 97 40–48. 10.1016/j.envexpbot.2013.10.001

[B44] Takahashi R. Bashir K. Ishimaru Y. Nishizawa N. K. Nakanishi H. (2012a). The role of heavy-metal ATPases, HMAs, in zinc and cadmium transport in rice. *Plant Signal. Behav.* 7 1605–1607. 10.4161/psb.22454 23072989 PMC3578901

[B45] Takahashi R. Ishimaru Y. Shimo H. Ogo Y. Senoura T. Nishizawa N. K. (2012b). The OsHMA2 transporter is involved in root-to-shoot translocation of Zn and Cd in rice. *Plant Cell Environ.* 35 1948–1957. 10.1111/j.1365-3040.2012.02527.x 22548273

[B46] Takahashi R. Ishimaru Y. Nakanishi H. Nishizawa N. K. (2011a). Role of the iron transporter OsNRAMP1 in cadmium uptake and accumulation in rice. *Plant Signal. Behav.* 6 1813–1816. 10.4161/psb.6.11.17587 22067109 PMC3329356

[B47] Takahashi R. Ishimaru Y. Senoura T. Shimo H. Ishikawa S. Arao T. (2011b). The OsNRAMP1 iron transporter is involved in Cd accumulation in rice. *J. Exp. Bot.* 62 4843–4850. 10.1093/jxb/err136 21697258 PMC3192999

[B48] Takahashi R. Ishimaru Y. Shimo H. Bashir K. Senoura T. Sugimoto K. (2014). From laboratory to field: OsNRAMP5-knockdown rice is a promising candidate for Cd phytoremediation in paddy fields. *PLoS One* 9:e98816. 10.1371/journal.pone.0098816 24901230 PMC4047016

[B49] Tang L. Mao B. Li Y. Lv Q. Zhang L. Chen C. (2017). Knockout of OsNramp5 using the CRISPR/Cas9 system produces low Cd-accumulating indica rice without compromising yield. *Sci. Rep.* 7:14438. 10.1038/s41598-017-14832-9 29089547 PMC5663754

[B50] Tezuka K. Miyadate H. Katou K. Kodama I. Matsumoto S. Kawamoto T. (2010). A single recessive gene controls cadmium translocation in the cadmium hyperaccumulating rice cultivar Cho-Ko-Koku. *Theor. Appl. Genet.* 120 1175–1182. 10.1007/s00122-009-1244-6 20039013

[B51] Toki S. Hara N. Ono K. Onodera H. Tagiri A. Oka S. (2006). Early infection of scutellum tissue with Agrobacterium allows high-speed transformation of rice. *Plant J.* 47 969–976. 10.1111/j.1365-313X.2006.02836.x 16961734

[B52] Ueno D. Koyama E. Yamaji N. Ma J. F. (2011). Physiological, genetic, and molecular characterization of a high-Cd-accumulating rice cultivar, Jarjan. *J. Exp. Bot.* 62 2265–2272. 10.1093/jxb/erq383 21127026

[B53] Ueno D. Yamaji N. Kono I. Huang C. F. Ando T. Yano M. (2010). Gene limiting cadmium accumulation in rice. *Proc. Natl. Acad. Sci. U.S.A.* 107 16500–16505. 10.1073/pnas.1005396107 20823253 PMC2944702

[B54] Uraguchi S. Kamiya T. Sakamoto T. Kasai K. Sato Y. Nagamura Y. (2011). Low-affinity cation transporter (OsLCT1) regulates cadmium transport into rice grains. *Proc. Natl. Acad. Sci. U.S.A.* 108 20959–20964. 10.1073/pnas.1116531109 22160725 PMC3248505

[B55] Uraguchi S. Sone Y. Ohta Y. Ohkama-Ohtsu N. Hofmann C. Hess N. (2017). Identification of C-terminal regions in *Arabidopsis thaliana* phytochelatin synthase 1 specifically involved in activation by arsenite. *Plant Cell Physiol.* 59 500–509. 10.1093/pcp/pcx204 29281059

[B56] Van Maele-Fabry G. Lombaert N. Lison D. (2016). Dietary exposure to cadmium and risk of breast cancer in postmenopausal women: a systematic review and meta-analysis. *Environ. Int.* 86 1–13. 10.1016/j.envint.2015.10.003 26479829

[B57] Vinceti M. Venturelli M. Sighinolfi M. C. Trerotoli P. Bonvicini F. Ferrari A. (2007). Case-control study of toenail cadmium and prostate cancer risk in Italy. *Sci. Total Environ.* 373 77–81. 10.1016/j.scitotenv.2006.11.005 17175009

[B58] von Zglinicki T. Edwall C. Ostlund E. Lind B. Nordberg M. Ringertz N. R. (1992). Very low cadmium concentrations stimulate DNA synthesis and cell growth. *J. Cell Sci.* 103 1073–1081. 1487490 10.1242/jcs.103.4.1073

[B59] Waisberg M. Joseph P. Hale B. Beyersmann D. (2003). Molecular and cellular mechanisms of cadmium carcinogenesis. *Toxicology* 192 95–117. 10.1016/s0300-483x(03)00305-6 14580780

[B60] Wang X. Liu Y. Zeng G. Chai L. Song X. Min Z. (2008). Subcellular distribution and chemical forms of cadmium in *Bechmeria nivea* (L.) Gaud. *Environ. Exp. Bot.* 62 389–395. 10.1016/j.envexpbot.2007.10.014

[B61] Watanabe S. Zhuo X. G. Kimira M. (2004). Food safety and epidemiology: new database of functional food factors. *Biofactors* 22 213–219. 10.1002/biof.5520220144 15630286

[B62] Weigel H. J. Jager H. J. (1980). Subcellular distribution and chemical form of cadmium in bean plants. *Plant Physiol.* 65 480–482. 10.1104/pp.65.3.480 16661218 PMC440359

[B63] Wu D. Sato K. Ma J. F. (2015). Genome-wide association mapping of cadmium accumulation in different organs of barley. *New Phytol.* 208 817–829. 10.1111/nph.13512 26061418

[B64] Xu Q. Wang C. Li S. Li B. Li Q. Chen G. (2017). Cadmium adsorption, chelation and compartmentalization limit root-to-shoot translocation of cadmium in rice (*Oryza sativa* L.). *Environ. Sci. Pollut. Res.* 24 11319–11330. 10.1007/s11356-017-8775-1 28303536

[B65] Yadav A. K. Shankar A. Jha S. K. Kanwar P. Pandey A. Pandey G. K. (2015). A rice tonoplastic calcium exchanger, OsCCX2 mediates Ca2+/cation transport in yeast. *Sci. Rep.* 5:17117. 10.1038/srep17117 26607171 PMC4660821

[B66] Yamaji N. Ma J. F. (2014). The node, a hub for mineral nutrient distribution in graminaceous plants. *Trends Plant Sci.* 19 556–563. 10.1016/j.tplants.2014.05.007 24953837

[B67] Yamaji N. Ma J. F. (2017). Node-controlled allocation of mineral elements in Poaceae. *Curr. Opin. Plant Biol.* 39 18–24. 10.1016/j.pbi.2017.05.002 28558362

[B68] Yamaji N. Takemoto Y. Miyaji T. Mitani-Ueno N. Yoshida K. T. Ma J. F. (2017). Reducing phosphorus accumulation in rice grains with an impaired transporter in the node. *Nature* 541:136. 10.1038/nature21404 28002408

[B69] Yamaji N. Xia J. Mitani-Ueno N. Yokosho K. Ma J. F. (2013). Preferential delivery of zinc to developing tissues in rice is mediated by P-type heavy metal ATPase OsHMA2. *Plant Physiol.* 162 927–939. 10.1104/pp.113.216564 23575418 PMC3668081

[B70] Yan J. Wang P. Wang P. Yang M. Lian X. Tang Z. (2016). A loss-of-function allele of OsHMA3 associated with high cadmium accumulation in shoots and grain of Japonica rice cultivars. *Plant Cell Environ.* 39 1941–1954. 10.1111/pce.12747 27038090

[B71] Yang A. Zhang W.-H. (2016). A small GTPase, OsRab6a, is involved in the regulation of iron homeostasis in rice. *Plant Cell Physiol.* 57 1271–1280. 10.1093/pcp/pcw073 27257291

[B72] Yang M. Zhang Y. Zhang L. Hu J. Zhang X. Lu K. (2014). OsNRAMP5 contributes to manganese translocation and distribution in rice shoots. *J. Exp. Bot.* 65 4849–4861. 10.1093/jxb/eru259 24963001 PMC4144776

[B73] Yang Y. Xiong J. Chen R. Fu G. Chen T. Tao L. (2016). Excessive nitrate enhances cadmium (Cd) uptake by up-regulating the expression of OsIRT1 in rice (*Oryza sativa*). *Environ. Exp. Bot.* 122 141–149. 10.1016/j.envexpbot.2015.10.001

[B74] Yokosho K. Yamaji N. Ma J. F. (2016). OsFRDL1 expressed in nodes is required for distribution of iron to grains in rice. *J. Exp. Bot.* 67 5485–5494. 10.1093/jxb/erw314 27555544 PMC5049396

[B75] Yuan L. Yang S. Liu B. Zhang M. Wu K. (2012). Molecular characterization of a rice metal tolerance protein, OsMTP1. *Plant Cell Rep.* 31 67–79. 10.1007/s00299-011-1140-9 21892614

[B76] Zhang X. Zhang M. Takano T. Liu S. (2011). Characterization of an AtCCX5 gene from *Arabidopsis thaliana* that involves in high-affinity K+ uptake and Na+ transport in yeast. *Biochem. Biophys. Res. Commun.* 414 96–100. 10.1016/j.bbrc.2011.09.030 21945443

