# Supplementary material for: A Node-Expressed Transporter OsCCX2 Is Involved in Grain Cadmium Accumulation of Rice
Source: Front Plant Sci. 2018 Apr 11;9:476. doi: 10.3389/fpls.2018.00476 (PMC5904359; doi:10.3389/fpls.2018.00476)
Supplement: Supplementary file 1 [file Table_1.PDF]

Table S1. Primer list for cloning and vector construction

Primer name	Primer Sequence (Sense 5’-3’)	Anti-sense 5’-3’
OsCCX2-Cas9-1	GGCAGTTTTATGGCGCTCCTGCGC	AAACGCGCAGGAGCGCCATAAAAC
OsCCX2-Cas9-2	GGCACCCTCGCCGCCTGACAATCC	AAACGGATTGTCAGGCGGCGAGGG
OsCCX2-Cas1JC	CGCGCACTACGGTACACGCA	ACGACGTGGCGAAGACAT
OsCCX2-Cas2JC	ATGTCTTCGCCAGCGTCGTCT	AGGAGGGCGCCGCCGAGGA
OsCCX2-p2300	CCCAAGCTTATGGCGCTCCTGCGCA	CGGGATCCCGTGCTGGCCACCAGGAG
OsCCX2-GUS	CCTCTAGATTATAATTTATTTTATCATG	TACCATG GAAAACGGGGGAGAACG
OsCCX2-qPCR	GTTCGTGTCCACCGTTGTT	TGGCGAGGAGTGAGCAGA
OsCCX2-YES2	CGGGATCCATGGCGCTCCTGCGCA	CTCTAGACTATGCTGGCCACCAGGAGT
