# Supplementary material for: A Node-Expressed Transporter OsCCX2 Is Involved in Grain Cadmium Accumulation of Rice
Source: Front Plant Sci. 2018 Apr 11;9:476. doi: 10.3389/fpls.2018.00476 (PMC5904359; doi:10.3389/fpls.2018.00476)
Supplement: Supplementary file 2 [file Table_2.PDF]

Table S2. Statistic of genetically modified offspring

	No. of T_0 Trangenic plants	No. of heterozygote	Variation type	No. of homozygote	Ratio of homozygote
ccx2-1	31	9	+A	8	51.6%
			+G	2	
			-G	6	
ccx2-2	21	9	-A	5	33%
			+A	2	
