# Supplementary material for: A Node-Expressed Transporter OsCCX2 Is Involved in Grain Cadmium Accumulation of Rice
Source: Front Plant Sci. 2018 Apr 11;9:476. doi: 10.3389/fpls.2018.00476 (PMC5904359; doi:10.3389/fpls.2018.00476)
Supplement: Supplementary file 3 [file Image_1.PDF]

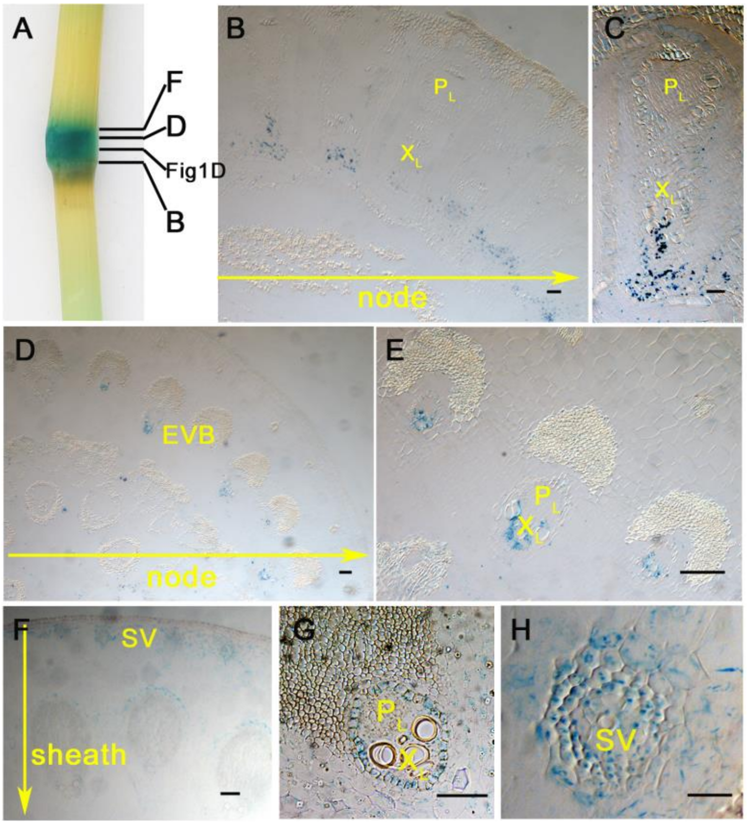

Fig S1. Paraffin section of different section in Node I. The GUS signal was shown in blue. Histochemical GUS staining of OsCCX2 pro:GUS plants. Bar=100 μm.

(A) Node I marked with B-F to show different levels from which cross slice were generated.

Images in (B), (D) and (F) showed the cross slices in order from bottom to top. Fig 1D refers to the images shown in figure 1D.

(B) Image of EVBs

(C) enlarged image of an EVB in (B).

(D) Image of EVBs in the sheath-node joint tissues.

(E) enlarged image of an EVB in (D).

(F) EVBs and small vascular tissues (SVs) in the sheath-node joint tissues.

(G) enlarged image of an EVB in (F).

(H) enlarged image of a SV in (F).
