# Supplementary material for: A Node-Expressed Transporter OsCCX2 Is Involved in Grain Cadmium Accumulation of Rice
Source: Front Plant Sci. 2018 Apr 11;9:476. doi: 10.3389/fpls.2018.00476 (PMC5904359; doi:10.3389/fpls.2018.00476)
Supplement: Supplementary file 4 [file Image_2.PDF]

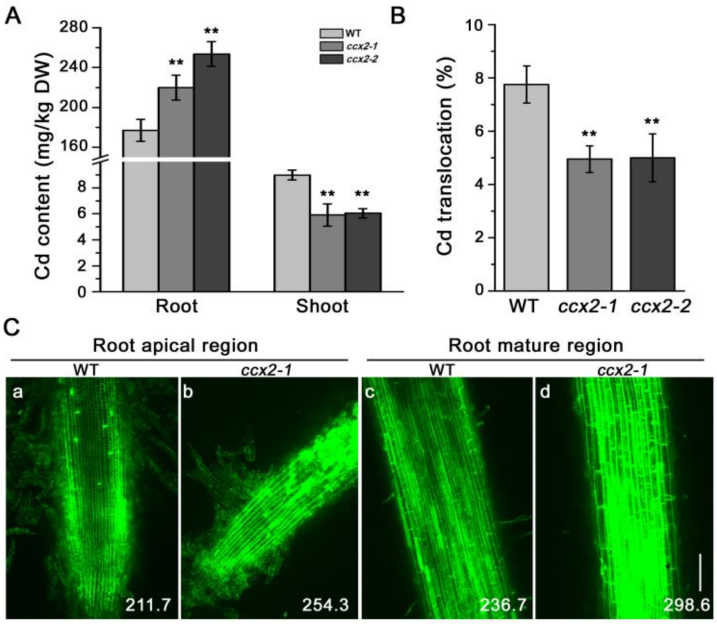

Figure S2. The root to shoot translocation of Cd.

10-day-old seedlings were transplanted to Cd-containing (5 μM) hydroponic solution for 7d. Cd content in root and shoot was measured (C), and Cd translocation ratio of shoot-to-root was calculated (D). Error bars represent ±SD of three biological replicates. Asterisks above the bars indicate significant difference (* P<0.05) compared with the WT rice.

A. Cd content in root and shoot of the seedlings.

B. Cd translocation ratio of shoot-to-root.

C. Detection of Cd in the root tips of rice by Leadmium™ Green AM fluorescent dye. The root tip tissues stained with Leadmium™ Green AM fluorescent dye for 2 h, and were analyzed by confocal microscopy using 488 nm excitation and measuring fluorescence emission at 520 nm. Bar =100 μm.
