# Supplementary material for: A Node-Expressed Transporter OsCCX2 Is Involved in Grain Cadmium Accumulation of Rice
Source: Front Plant Sci. 2018 Apr 11;9:476. doi: 10.3389/fpls.2018.00476 (PMC5904359; doi:10.3389/fpls.2018.00476)
Supplement: Supplementary file 5 [file Image_3.PDF]

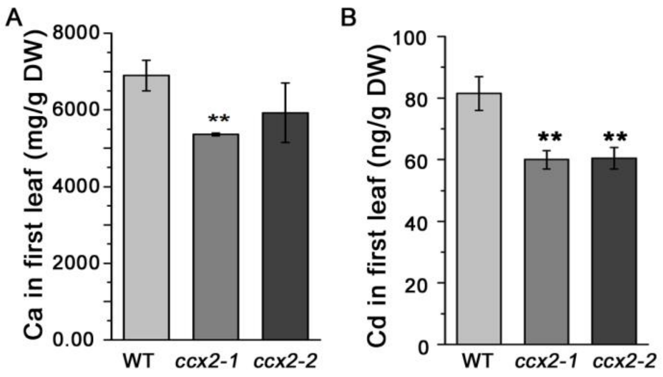

Fig S3. Cd and Ca content assays in the first leaf at the grain-filling initiation stage. The *ccx2-1*, *ccx2-2* mutant lines and the wild type control were grown in Cd-containing paddy soil (1.2 mg/kg) till grain-filling initiation, then the xylem sap was collected from a cut in the middle of the uppermost internode. Error bars represent ±SD of three biological replicates. Asterisks above the bars indicate significant differences (** P<0.01) compared with the WT rice.

A. Ca content in the first leaf

B. Cd content in the first leaf
